# Deep hydrous melting of the mantle wedge recorded by Mg isotopes

**DOI:** 10.1126/sciadv.aei6572

**Published:** 2026-10-14

**Authors:** Winnie X. Fan, Fang-Zhen Teng, Yinbiao Peng, Anna D. Berg, William P. Leeman

**Affiliations:** ^1^ Isotope Laboratory, Department of Earth and Space Sciences, University of Washington, Seattle, WA 98195, USA.; ^2^ Department of Earth and Planetary Sciences, Harvard University, Cambridge, MA 02138, USA.; ^3^ Department of Earth and Environmental Science, Rice University, Houston, TX 77005, USA.

## Abstract

Subduction zones govern Earth’s deep volatile cycle. The slab-mantle interface is a key locus of volatile exchange between the subducting crust and the overlying mantle wedge, yet whether and how chemical reactions at this interface affect arc magmatism remains poorly constrained. Arc magmas typically lack a geochemical hallmark of garnet fractionation, leading to the prevailing interpretation that they derive predominantly from the shallower, garnet-free mantle wedge. Here, we challenge this view using Mg isotopic data from Nicaragua, the South Sandwich Islands, and the central-eastern Aleutian arc, integrated with a global arc magma compilation. We show that heavy Mg isotopic signatures in mafic arc magmas vary with indicators of volatile enrichment, pointing to a volatile-rich source, most likely the heavily metasomatized base of the mantle wedge. The heaviest Mg isotopic compositions occur in arcs underlain by the most steeply dipping subducting slab, suggesting a focusing effect by the geometry of the downgoing slab. Monte Carlo isotope mixing models indicate that up to 50% of the Mg budget in some arc magma sources derives from deep, water-saturated melting near the slab-mantle interface. These findings highlight the base of the mantle wedge as a volumetrically and geochemically significant contributor to arc magmatism and the water cycle.

## INTRODUCTION

Subduction processes play a critical role in modulating the Earth’s surface and deep volatile cycles, including its water budget. As the most abundant volatile in the mantle wedge, water also plays a critical role in driving melt production at magmatic arcs (*1*, *2*). As water released from the downgoing slab can drastically lower the peridotite solidus temperature, melting in subduction zones can be initiated near the base of the mantle wedge under water-saturated conditions (*3*, *4*). Such hydrous initial melts (Type 1) may migrate upward and promote water-undersaturated melting (Type 2) in the hotter interior of the mantle wedge. Derived from the heavily metasomatized base of the mantle wedge, this initial low-degree, water-saturated melt can dominate the trace element and volatile budget of the erupted arc magmas.

Both types of mantle melting are supported by experimental and geodynamic studies, yet their relative contributions to arc magmas remain poorly constrained. England & Katz (*5*, *6*) suggested that melt production is dominantly controlled by the hottest part of the mantle wedge above the anhydrous peridotite solidus (Type 2), whereas Grove *et al.* (*7*) emphasize the importance of breakdown of hydrous minerals triggering water-saturated peridotite melting near the base of the mantle wedge (Type 1). Distinguishing these melting regimes in natural samples is difficult by trace elements since garnet is largely consumed during partial melting of garnet peridotites within ∼100°C of water-saturated solidus (*3*, *4*). Thus, deep-seated Type 1 melt does not necessarily record canonical garnet signatures (e.g. high La/Yb, Sr/Y). Major elemental compositions can provide some insights. Experimental studies suggest that some primitive andesites and basaltic andesites may incorporate 12–45% melts derived from deep, hydrous melting near the base of the mantle wedge (*8*). However, erupted arc magmas, including mafic magmas, generally undergo some degree of crystallization, obscuring their primary major element compositions and limiting applications of this approach (*9*).

Magnesium isotope systematics provide a promising tool for quantifying this mass balance and can be applicable beyond near-primary melts. Due to the limited fractionation during mantle melting and basaltic differentiation (*10*), arc magmas largely preserve the isotopic composition of their parent melts and, ultimately, their mantle sources. While the upper subarc mantle wedge has Mg isotopic compositions indistinguishable from the mantle elsewhere (*11*), the metasomatized ultramafic rocks at the base of the mantle wedge can be enriched in heavy Mg isotopes due to reactions with slab-derived fluids and/or melts (Fig. 1B; *12*–*14*). Consequently, contributions from different parts of the mantle wedge can generate arc magmas with a range of Mg isotopic compositions across volcanic arcs.

**Fig. 1.**
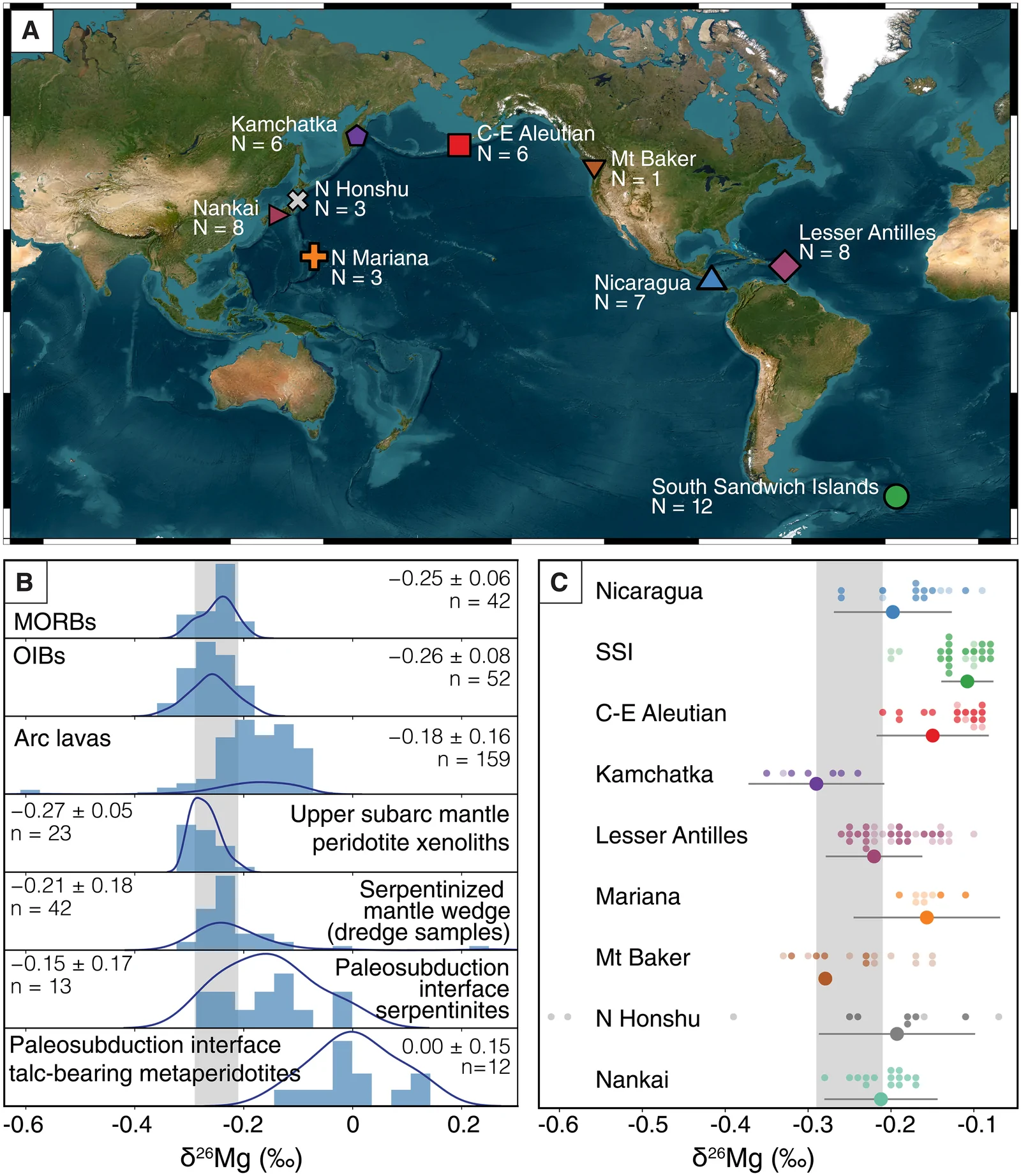
Magnesium isotopic systematics of arc lavas, other mantle-derived melts, and variably metasomatized mantle samples. (**A**) Global distribution of Quaternary arc magma samples with published Mg isotopic data, including the new data reported in this study. N refers to the number of volcanic centers with samples containing ≥5 wt.% MgO. (**B**) δ^26^Mg variations in mantle-derived melts and variably serpentinized subarc mantle. Mid-ocean ridge basalts (MORBs) and oceanic island basalts (OIBs) data are from (*76*); peridotite wedge mantle data are from (*11*, *37*); serpentinized wedge mantle are from (*37*, *38*). (**C**) δ^26^Mg variations in Quaternary arc lavas from 9 arc segments (data S1). Within each arc segment, darker/bolder color denotes samples with ≥5 wt % MgO and lighter/muted color denotes samples with <5 wt % MgO. Large dots with error bars represent the average and 2SD of δ^26^Mg for each arc segment by first averaging high MgO samples from each volcanic center. Nicaragua, SSI, and C-E Aleutian data are from this study and are reported in table S1. Compilation of literature data is available in data S1. Kamchatka data are from (*19*); Northern Mariana data are from (*18*); Lesser Antilles data are from (*17*, *18*); Mt. Baker data are from (*55*); Northern Honshu and Nankai data are from (*77*). Grey shaded band in (B) and (C) represent the range of δ^26^Mg for the mantle (−0.25 ± 0.04‰) (*10*). Basemap is World Imagery by Esri (*78*). Map image is the intellectual property of Esri and is used herein under license. Copyright 2025 Esri and its licensors. All rights reserved.

Mafic arc magmas are typically ∼0.1 to 0.15‰ heavier than the pristine mantle [δ^26^Mg = −0.25 ± 0.04‰, 2SD (*10*)], whereas MORBs and OIBs largely overlap with mantle values (*10*, *15*). In this work, we propose that this small yet resolvable enrichment records contributions from the metasomatized mantle wedge near the slab-mantle interface (i.e., Type 1 melting). Specifically, we hypothesize that variations in the Mg isotopic compositions of arc magmas reflect differences in the relative contributions of Type 1 and Type 2 melts. To explore this model, we report Mg isotopic compositions of Pliocene-Quaternary arc basaltic to basalt andesitic lavas from the South Sandwich Islands, Nicaragua, and the central-eastern Aleutian arc, and compare them with literature data (Fig. 1). This dataset pertains to a wide range of slab thermal parameters, enabling examination of melt generation and associated Mg isotopic variations across subduction regimes. We show that high δ^26^Mg values in arc magmas are linked to trace element signatures of flux melting (e.g., relative enrichment of fluid-mobile elements) and is most pronounced in arcs with intermediate thermal conditions and steep dipping subducting slab, suggesting a slab-controlled focusing effect of metasomatism. Monte Carlo mixing models indicate up to 50% of arc magma may originate from deep, water-saturated melting near the slab-mantle interface. The prevalence of high δ^26^Mg in arc lavas therefore indicates the base of the mantle wedge plays a major role in melt generation and water transfer.

## RESULTS

Magnesium isotopic compositions (δ^26^Mg, where higher values indicate enrichment in heavy isotopes) and MgO contents for all samples are presented in table S1 and summarized below.

The δ^26^Mg values of the 14 Nicaragua samples range from −0.26‰ to −0.09‰ (mean = −0.16‰ ± 0.04‰, 2SD). Their MgO content ranges from 2.2 to 11.02 wt %. These mostly basaltic samples display a moderate negative correlation between δ^26^Mg and MgO content, as well as Mg# (Fig. 2 and fig. S1), driven largely by three high MgO Central Nicaragua samples with lower δ^26^Mg values: NE203 (−0.26‰ ± 0.04‰, MgO = 11.02 wt %), GR5 (−0.21‰ ± 0.09‰,), and TE6 (−0.26‰ ± 0.04‰). The remaining samples define a relatively narrow range of −0.21‰ to −0.09‰ with an average of −0.15 ‰ ± 0.07 ‰.

**Fig. 2.**
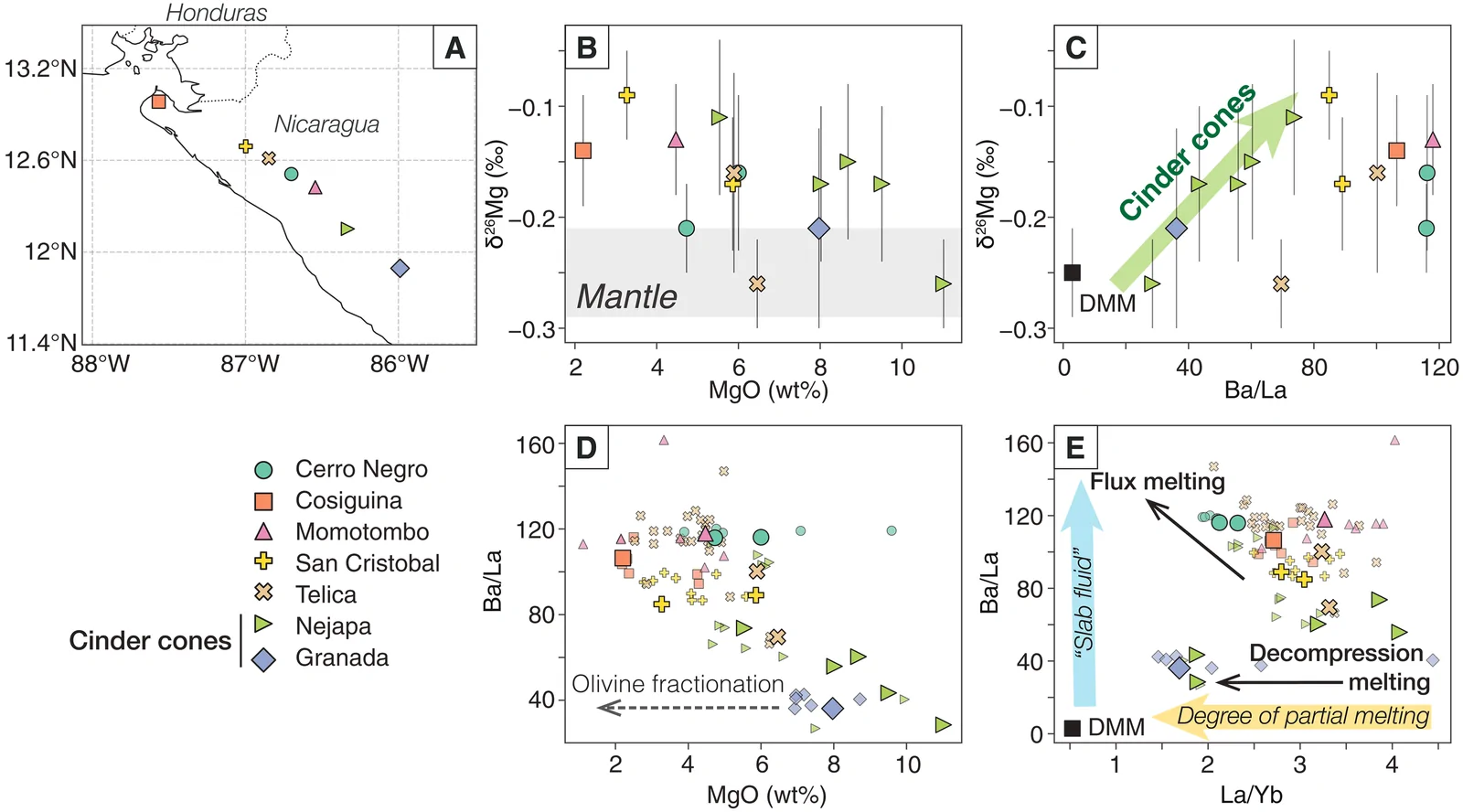
Magnesium isotopic systematics of Nicaragua arc lavas. (**A**) Map showing the location of volcanic centers sampled in this study. (**B**) Plot of δ^26^Mg vs. MgO content, (**C**) δ^26^Mg vs. Ba/La, (**D**) Ba/La vs. MgO content, and (**E**) Ba/La vs. La/Yb. While the moderate correlation between δ^26^Mg vs. MgO content points to apparent effect of olivine fractionation, the trace element systematics suggest the variation of Mg isotopic composition is best explained by different drivers for melt production. Larger opaque symbols in panels D to E correspond to Mg isotope data, smaller translucent symbols are data from the RU_CAGeochem database (*65*) that were not analyzed for Mg isotopes. Depleted MORB mantle (DMM) trace element composition is from (*79*); DMM and mantle Mg isotopic composition is from (*10*).

The South Sandwich Islands samples (basalts to andesites) have δ^26^Mg of −0.20‰ to −0.08‰ (mean = −0.12‰ ± 0.07‰, 2SD), MgO of 2.46 to 10.95 wt %, and Mg# of 27.9 to 61.2, indicating diverse magmatic processes across the arc. These data are consistent with those reported by Qiao *et al.* (*16*).

The central-eastern (C-E) Aleutian samples (all basaltic) have δ^26^Mg of −0.12‰ to −0.06‰ (mean = −0.13‰ ± 0.08‰, 2SD) and MgO of 4.05 to 8.7 wt %.

Overall, these arc lavas have δ^26^Mg ∼ 0.1–0.15‰ higher than the typical mantle range, consistent with previously published Quaternary arc basalts (*16*–*19*) (Fig. 1C). For geodynamic models of modern subduction zones to be relevant, our compilation includes samples of Pliocene-Quaternary in age. These samples span 45 to 64 wt % SiO_2_ and 1.3 to 17.3 wt % MgO with predominantly basaltic compositions. Although high- and low-MgO groups have no systematic difference in δ^26^Mg, significant inter-arc variation is observed (Fig. 1C).

## DISCUSSION

Equilibrium olivine-melt fractionation has been proposed to explain the high δ^26^Mg of arc lavas, based on an olivine-melt fractionation factor Δ^26^Mg_olivine-melt_ = ∼0.07‰ constrained from natural olivine-melt inclusion pairs (*18*). However, Mg isotopic composition generally displays little systematic variation with MgO content in arc magmas, creating a discrepancy between crystal-scale and whole-rock records. Several factors could give rise to this discrepancy. The Mg-Fe interdiffusion between high Mg# crystals and low Mg# melts can produce olivine grains either heavier or lighter than the melt, but this process likely averages out at the whole rock scale, as observed in Kilauea Iki basalts (*10*, *20*). Constraining Δ^26^Mg_olivine-melt_ directly using melt inclusion-olivine pairs (*18*) can also be problematic as quenching of melt inclusions may induce Soret fractionation due to thermal diffusion (*18*, *21*), and boundary layer effects could make melt inclusions unrepresentative of bulk melt (*22*). While olivine-melt fractionation may partially contribute to the high δ^26^Mg values of arc magmas, it is hard to tease out its role from mantle source heterogeneity given the current uncertainty surrounding Δ^26^Mg_olivine-melt_. While the role of olivine fractionation remains uncertain, Mg isotopic compositions are known to be sensitive to crystallization of Mg-bearing oxides in evolved magmas (<5 wt % MgO) (*23*, *24*). Therefore, we filter Mg isotope data to only include samples with >5 wt % MgO when plotting with trace elements and calculating arc averages. The Mg isotopic variation in these filtered high MgO arc magmas likely reflects characteristics of their mantle sources, as discussed below.

### Enrichments of heavy Mg isotopes and fluid mobile elements in Central Nicaragua and beyond

In our new data, Mg isotope compositions of samples from C-E Aleutian and South Sandwich Islands display no correlation with MgO content and molar Mg# (fig. S1), suggesting that variations of δ^26^Mg are unlikely related to basalt differentiation. Unlike C-E Aleutian and South Sandwich Islands, Nicaragua samples exhibit moderate negative correlation between δ^26^Mg, MgO content, and molar Mg# (fig. S1). This correlation is most obvious in samples from cinder cones of Nejapa and Granada alignments (Fig. 2, A and B). We analyzed 6 cinder cone samples ranging from 5.54–11.02 wt % MgO. These samples are more primitive compared to other magmas in Nicaragua, and likely approach the composition of a parental basaltic magma in this part of Nicaragua (*25*, *26*). With δ^26^Mg ranging from −0.26 ± 0.04 to −0.11 ± 0.07‰, they largely cover the full range of Mg isotope variation in Nicaragua arc magmas. Although this correlation seems to suggest that olivine fractionation drives Mg isotopic composition of the melt to be more enriched in heavy Mg, the high MgO and low δ^26^Mg samples cannot be related to other Nejapa samples solely by olivine fractionation, since they are also characterized by lower Ba/La and higher La/Yb ratios (Fig. 2C).

The covariations between Mg isotopes and trace elements in the Central Nicaraguan cinder cones are best explained by mixing of a flux melting product with a decompression driven melt (*26*) (Fig. 2E). Tectonically, Central Nicaragua is an area with active extension, best exemplified by the Managua Graben. The Nejapa volcanic field is situated on the N-S striking Nejapa fault, marking the western active edge of the Managua Graben (*27*). Extension in the overlying crust could drive decompression melting of the mantle wedge, and the lack of a large magma chamber and thick overlying crust at these monogenetic vents allow the geochemistry of both endmember melts to be displayed in the erupted products with minimal modification by crustal processes (*28*).

The more primitive Nejapa-Granada cinder cone samples, characterized by mantle-like Mg isotopic compositions and the lack of FME enrichments compared to other Nicaragua samples, were attributed to decompression melting (Fig. 2). Similar magmas are also observed in Kamchatka and Mt. Baker (Fig. 3), where decompression melting could play an important role. Experimental petrology constraints suggest that primitive melts from Kluychevskoy, Kamchatka are products of lherzolite melting driven by rifting-induced decompression (*29*). Upwelling of enriched asthenosphere mantle through the slab edge has also been proposed to account for the occurrence of OIB-like geochemical characteristics in some North Cascadia arc magmas (*30*). In these instances, the mantle-like Mg isotopic compositions of arc magmas are indicators of predominantly decompression-driven melting.

**Fig. 3.**
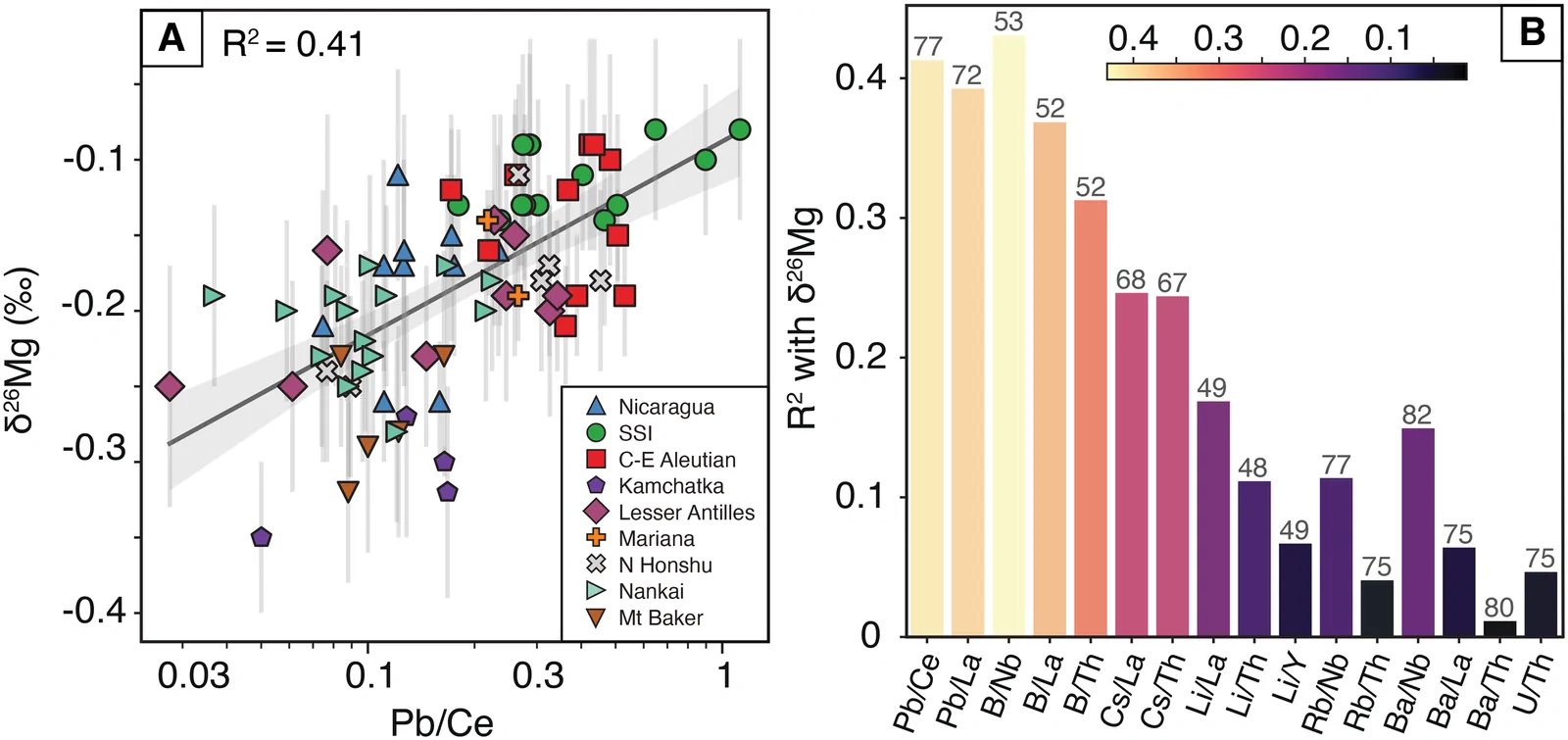
Relationship between high δ^26^Mg and FME enrichment in global arc lavas. (**A**) Plot of δ^26^Mg versus Pb/Ce of arc lava samples with MgO content >5 wt %. Black line and shaded area represent results of logarithmic ordinary least square regression and its 95% confidence interval. (**B**) Bar plot showing the R^2^ values from logarithmic regressions between δ^26^Mg and selected trace element ratios. The elements in the numerator are commonly referred to as fluid mobile elements [FMEs; though the inclusion of Li and Cs varies between authors, and some authors include U and Ba. Leeman first defined FMEs to be B, As, Sb, and Pb (*80*)]. Numbers above each bar indicate the number of samples used in the regression with available trace element data.

Globally, high δ^26^Mg is also linked to enrichment of fluid mobile elements (FMEs) characteristic of flux melting (Fig. 3). Slab-derived fluid contributions are implicated by enrichments of FMEs relative to fluid-immobile elements of similar magmatic compatibility (e.g., Pb/Ce, Ba/La, B/Nb), ratios that are relatively insensitive to magmatic differentiation. Among these proxies, δ^26^Mg correlates more strongly with Pb/Ce than Ba/La, and displays moderate correlations with ratios involving other FMEs such as B, Li, Pb, and Cs (Fig. 3). FMEs are highly soluble in slab-derived fluids even at lower P-T conditions and are released early during slab dehydration (*31*), as reflected by their enrichment in subduction zone serpentinites (*32*) and strong across-arc variations in erupted magmas (*31*). Consequently, as slab-derived fluids react with the overlying mantle, the base of the mantle wedge becomes strongly enriched in FMEs (*32*, *33*). However, the coupled enrichment of FMEs and high δ^26^Mg is unlikely to result from dehydration of serpentinized mantle wedge since Mg mobility in aqueous fluids is limited relative to FMEs, especially at forearc P-T conditions. By contrast, a melt phase can efficiently mobilize both Mg and incompatible FMEs, making deep, volatile-fluxed melting near the slab-mantle interface the more viable source. Therefore, arc magma δ^26^Mg could be used to discern the contribution of deep hydrous melting versus decompression melting in the hot interior of the mantle wedge.

### Hydrous melting near the base of the mantle wedge

A common explanation for the high δ^26^Mg of arc lavas invokes dehydration of slab interior serpentinites, which releases high δ^26^Mg fluids (*11*, *17*, *34*), based on the elevated δ^26^Mg values of abyssal serpentinites (*35*). These fluids metasomatize the mantle wedge, producing a high δ^26^Mg mantle source recorded in arc magmas (*36*). While this model can explain correlations between Mg isotopes and FMEs, direct evidence from subarc mantle xenoliths and wedge serpentinites does not support pervasive enrichment of heavy Mg isotopes in most of the mantle wedge. Kamchatka harzburgite xenoliths bear ubiquitous textural and trace element evidence of fluid metasomatism yet have mantle δ^26^Mg (*11*). Forearc serpentinites dredged from the seafloor are likewise mostly within the mantle range, with only few high δ^26^Mg outliers attributed to seafloor alteration (*16*, *37*, *38*). Exhumed serpentinized mantle wedge peridotites from the Franciscan Complex have close to mantle Mg isotopic compositions (*13*). This lack of isotopically heavy Mg is unlikely a result of melt extraction, as melt removes only a minor fraction of Mg from peridotite (*37*). The discrepancy between high δ^26^Mg arc samples and typical mantle wedge samples requires additional processes to bridge this isotopic gap and generate the isotopically heavy arc magmas.

We attribute this discrepancy to differences between near-slab (Type 1) and shallower (Type 2) melt source domains. The high δ^26^Mg values of arc magmas record the initial water-saturated melting near the base of the mantle wedge, where interactions with slab-derived fluids and/or melts enrich the local mantle in heavy Mg. While the upper subarc mantle retains typical mantle Mg isotopic compositions (*11*), the slab-mantle interface experiences intense fluid-rock interaction with significant Mg mobility (Fig. 4). This is well documented in exhumed paleo-subduction interfaces where fluid-driven reactions between mantle wedge serpentinite and the subducting slab preferentially remove light Mg isotopes from serpentinite, producing talc-, chlorite-, and actinolite-rich metasomatic mélange matrix (*12*–*14*, *39*). Specifically, formation of talc has been demonstrated to be a direct consequence of removal of Mg (with or without addition of Si) from the ultramafic lithologies during fluid exchange between the slab and the forearc mantle, with peak metamorphic conditions estimated to be ∼550°C and ∼ 1.2 GPa (*12*). The resulting talc-bearing ultramafic rocks reach δ^26^Mg values approaching 0‰ (Fig. 1B), and this heavy isotopic signature reflects either kinetic fractionation during fluid-driven diffusion or Rayleigh distillation via fluid-talc Mg isotopic partitioning (Fig. 4 inserts; *9*–*11*), with the complementary isotopically light Mg deposited in chlorite, actinolite, and other metasomatic minerals in mafic and metasedimentary lithologies (Fig. 4 inserts; *9*, *11*). Notably, these mechanisms for enriching the mantle wedge in heavy Mg isotopes are not particularly restrictive in terms of P-T conditions. Kinetic fractionation operates as long as fluids are present at the interface, meanwhile talc remains stable up to ∼2 GPa in the forearc (*40*) and is widely documented in paleosubduction interfaces (*12*). Accordingly, enrichment of the base layer of the mantle wedge in heavy Mg isotopes may be a common feature of both hot and cold subduction zones.

**Fig. 4.**
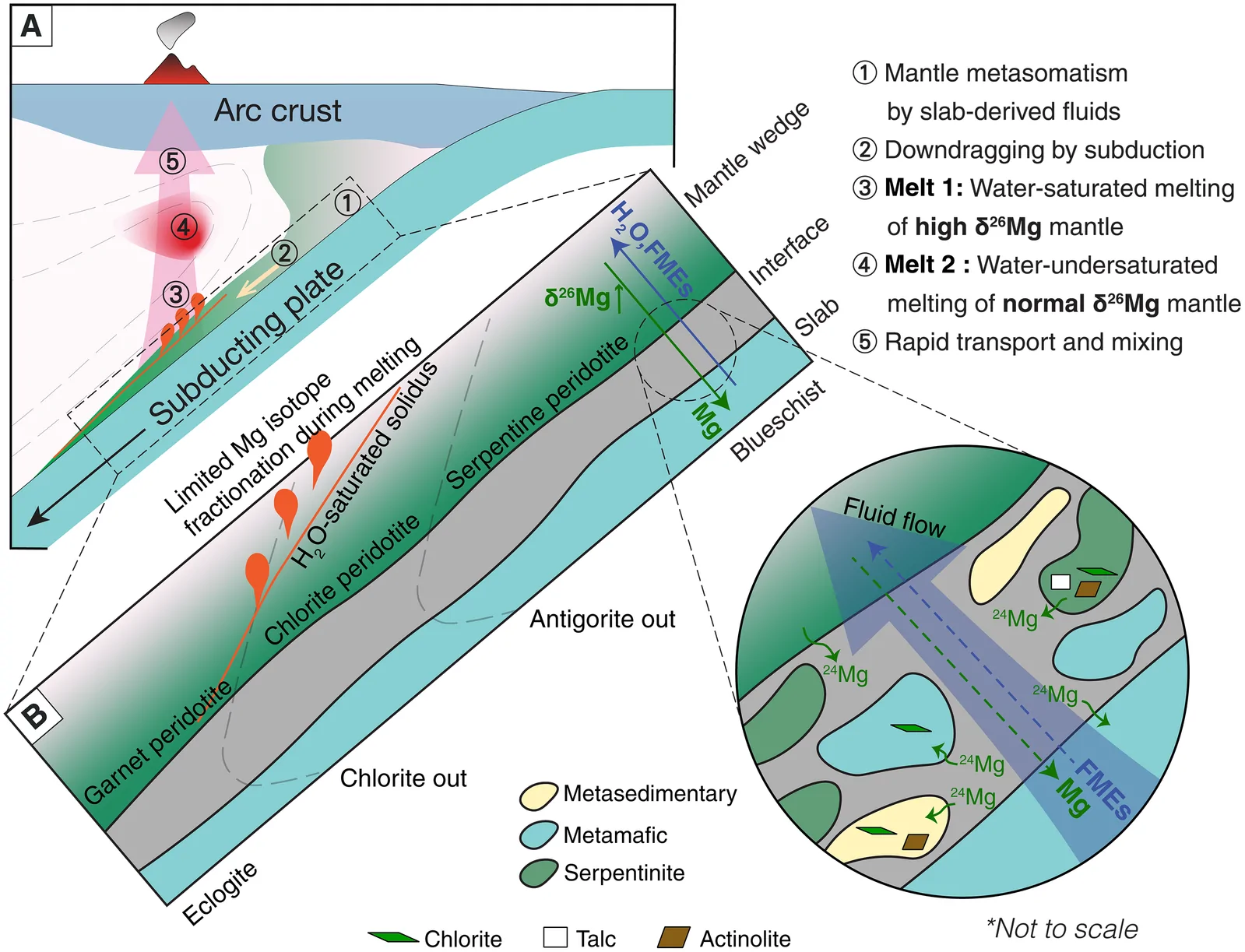
Initiation of hydrous melting in the deep metasomatized mantle wedge with high δ^26^Mg. (**A**) Schematic cross section of the mantle wedge illustrating the proposed sequence of processes leading to high δ^26^Mg in erupted mafic arc magmas: (1) Metasomatic exchange with the slab depletes Mg from the mantle while preferentially removing light Mg isotopes; (2) Continued subduction entrains this metasomatized domain to greater depths; (3) Initiation of Type 1 melting when this parcel of mantle intersects the water-saturated peridotite solidus; (4) Water-undersaturated melting in the warmer interior of the mantle wedge generating Type 2 melt; and (5) Chemically isolated transport and mixing of the two melt components. Insert panel (**B**) t demonstrates that the close proximity of chlorite breakdown condition and water-saturated peridotite solidus allows mantle melting to initiate near the base of the mantle wedge (*3*, *8*). Lithological transition is from (*41*, *81*) with the water-saturated peridotite solidus by (*3*). Zoom in circle shows the direction of fluid facilitated chemical exchange of FME and Mg accompanied by preferential loss of light Mg isotopes (^24^Mg) from the mantle, thus generating a high δ^26^Mg signal in serpentinized parts of the basal mantle wedge. The gray matrix can be talc-, chlorite-, or actinolite-rich metasomatic product from ultramafic or crustal lithologies, as well as their mechanical mixtures (*12*). The isotopic exchange during the metasomatic reaction is documented in exhumed paleosubduction complexes (*12*–*14*).

Although heavy Mg enrichment at the base of the mantle wedge requires fluid facilitation, the apparent fluid mobility of Mg differs fundamentally from that of FMEs. Efficient redistribution of Mg at the slab-mantle interface is sustained by the precipitation of secondary Mg-bearing minerals that maintain a chemical gradient for continued Mg transfer, which is likely a kinetic process (*14*). Consequently, fluids released by dehydration of mantle wedge serpentinite are unlikely to efficiently metasomatize the overlying mantle wedge with isotopically heavy Mg due to the lack of strong chemical gradient. By contrast, fluid-mobile elements are readily partitioned into the fluid phase even under lower pressure–temperature forearc conditions, and they are compatible in hydrous phases such as antigorite or chlorite during mantle hydration (*32*, *41*). Therefore, the stronger correlations between δ^26^Mg and highly mobile FMEs such as B and Pb, relative to the less mobile Ba and U (Fig. 3), suggest that the coupled enrichment of heavy Mg and FMEs is a cumulative effect of metasomatism, which initiates at the interface beneath the forearc and is inherited by the mantle wedge during subsequent subduction (Fig. 4).

Talc is stable only to ∼2 GPa (*40*), so the metamorphic record does not extend to subarc P-T conditions. At subarc depths, chlorite is the more likely hydrous phase in the mantle wedge, remaining stable under subsolidus conditions up to 5 GPa (*3*, *42*). While low-δ^26^Mg chlorites occur at subduction interfaces, they are derived from mafic protoliths and represent a sink for the light Mg lost from serpentinite during talc formation (*12*, *14*). No Mg isotopic data exist for high-Mg chlorite in metaperidotites, but the same processes that produce high δ^26^Mg in talc should apply during chlorite formation, given their similar fluid-mineral Mg isotope partitioning coefficients (*36*) and potentially even larger kinetic effects at higher temperature. Therefore, the isotope fractionation recorded in talc can serve as a proxy for deeper interface processes involving chlorite, indicating an isotopically heavy base of the mantle wedge at subarc depths (Fig. 4).

The base of the mantle wedge thus can transfer heavy Mg to subarc depths in chlorite, a significant host for water and FMEs (*41*, *43*). Chlorite dehydration at subarc depths triggers water-saturated melting, producing melts enriched in both heavy Mg and FMEs. This mechanism of melt generation has been hypothesized in previous works (*4*, *8*, *44*–*48*), and seismic observations also support the presence of serpentinized mantle near the subduction interface at subarc depths (*49*). Experimental studies further demonstrate that hydrous mantle melting can generate primary basaltic andesites and andesites (*46*–*48*). Consistent with this, the few available δ^26^Mg data of high-Mg andesites from East Junggar reach ∼+0.1‰ (*50*), indicating a dominant contribution from water-saturated melting near the base of the mantle wedge. Magnesium isotopes therefore provide direct evidence for the contribution of deep hydrous melt to erupted arc magmas.

### Subduction geometry, hydrous melting and melt transport

Variations in Mg isotopic composition between arcs reflect different proportions of near-slab melt (Type 1) vs. shallower mantle wedge melts (Type 2). To quantify the Type 1 melt contribution, we perform Monte-Carlo simulations of binary mixing between these two types of melts (Fig. 5; fig. S2; see Materials and Methods). The model assumes no post-generation modification of the deep melt endmember, making it a conservative estimate since any interaction with the overlying mantle wedge would drive Type 1 melts towards mantle δ^26^Mg. While the model describes mixing as binary, it does not distinguish true two-endmember melt mixing from progressive dilution of the original high-δ^26^Mg melts by mantle-like Mg. Our results indicate that despite appearing volumetrically minor, the metasomatized base of the mantle wedge supplies 20–50% of the Mg budget to mafic arc magmas. Through continuous subduction and interaction with the subducting slab, the high-δ^26^Mg base of the mantle wedge is persistently replenished, maintaining a geochemically distinct and sustained contribution to arc magmatism.

**Fig. 5.**
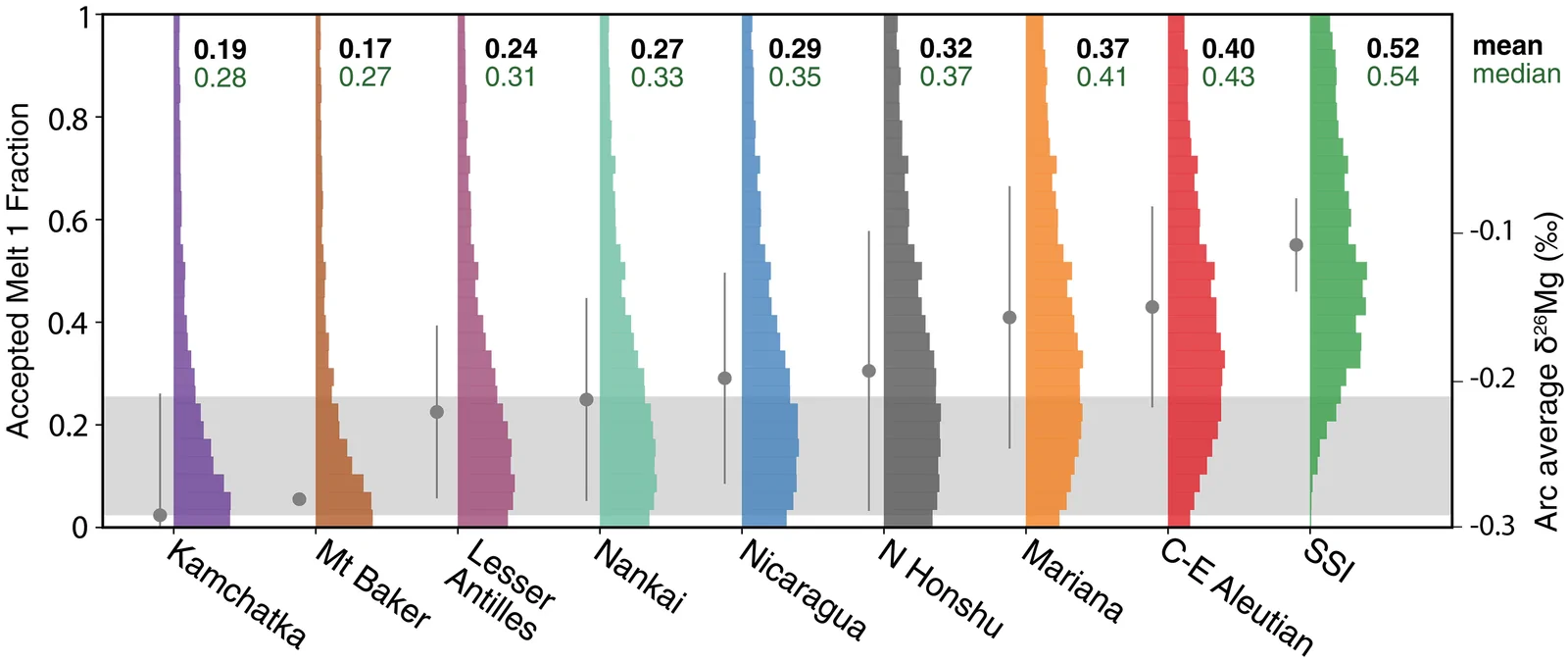
A major contribution of deep hydrous melting (Type 1) to arc magmatism. Histograms showing ranges of permissible mixing fractions inverted by comparing the δ^26^Mg of simulated melt to the average δ^26^Mg of each arc segment and filtered by SiO_2_ < 52 wt %. Histograms showing the distribution of the modeled fractions of Type 1 melt required to reproduce the average δ^26^Mg in the range of 2SD of each volcanic center, except for Mt. Baker where a range of ±0.05‰ is imposed to account for analytical uncertainty. The numbers near top of each histogram display the mean and median of permissible fractions of Type 1 melt for each arc. Gray dots with error bar show calculated average δ^26^Mg of arc lavas from the corresponding subduction zone. See Materials and Methods section for details.

Our results, based on the highly compatible element Mg, complement studies that use trace elements and radiogenic isotopes to trace the slab-derived components (e.g. *51*, *52*). Experimental studies demonstrate that <5% slab-derived material in the Type 1 source is sufficient to account for the enriched alkali and REE signatures of primitive basaltic andesites (*8*). Because Type 1 melts are generated from a more fertile mantle source and likely at lower degree of partial melting compared to Type 2 melts, they can dominate the incompatible element and isotopic signatures of final erupted arc magmas despite only modest slab addition to the base of the mantle wedge. Therefore, constraints by Mg isotopes on the relative mass contributions of these two melting regimes provide a complementary framework that links melt proportions to chemical signatures recorded by incompatible elements and isotopes.

To explore how subduction thermal structure (hot vs. cold) controls the relative contributions of the two melting mechanisms, we plot the calculated average δ^26^Mg against the slab thermal parameter ϕ=t v sinθ, where *t* is age of subducting plate at trench, *v* is the convergence rate, and θ is the slab dip angle (*53*). Arc δ^26^Mg displays a first order dependence on slab thermal parameter, peaking at intermediate values (Fig. 6A), which suggests that intermediate subduction systems represent a thermal ‘sweet spot’ for the greatest contribution of Type 1 melt.

**Fig. 6.**
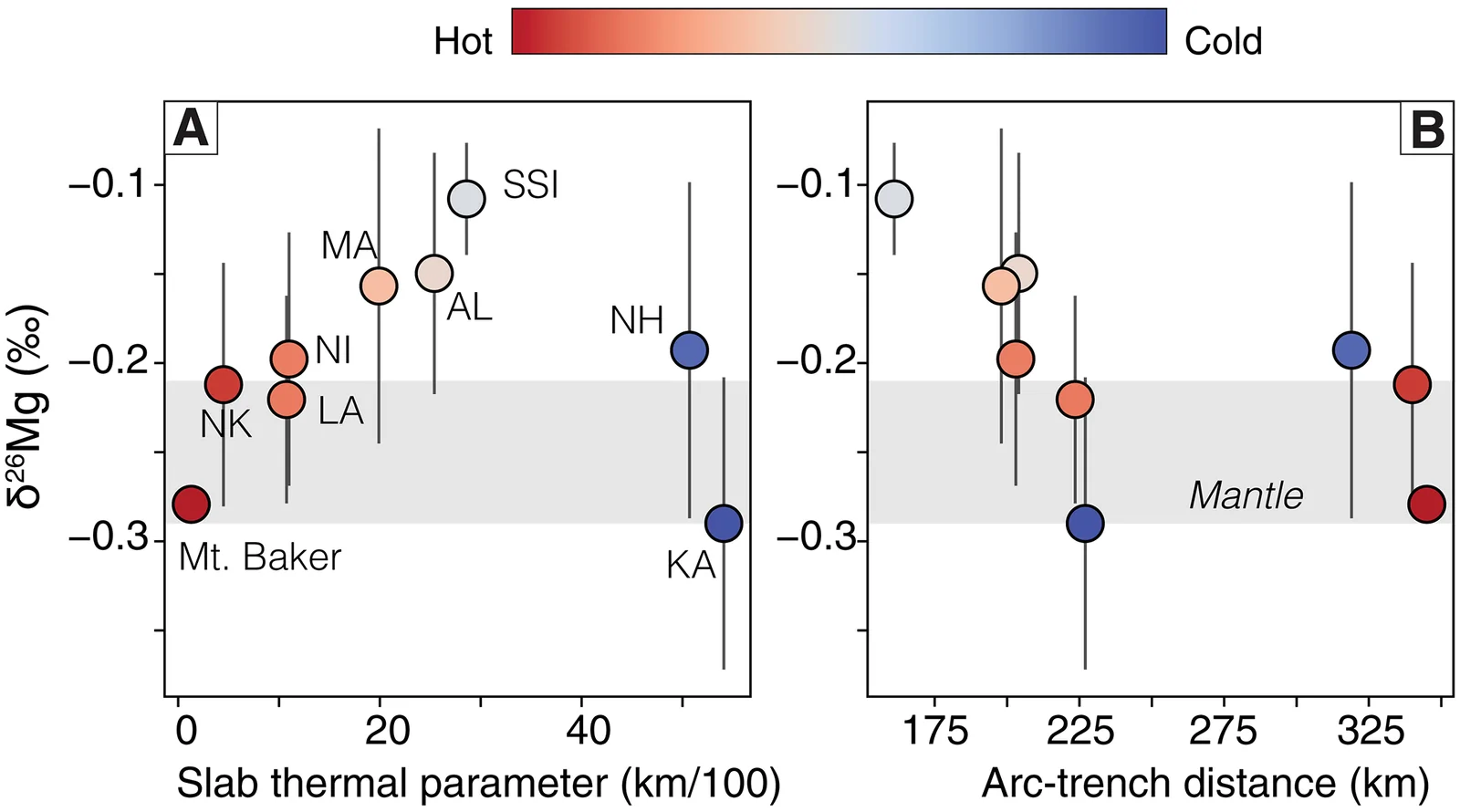
The heaviest arc magmas are from subduction zones with intermediate slab thermal condition and the steepest geometry. Average δ^26^Mg of arc lavas from each subduction zone plotted against (**A**) thermal parameter and (**B**) arc-trench distance. Average δ^26^Mg value of each subduction zone was calculated by first averaging each volcanic center. Color bar also represents the thermal parameter, where warm color corresponds to hot subduction (i.e. smaller thermal parameter), and vice versa. Thermal parameter and arc-trench distance were from (*53*, *77*) (table S3). Mantle Mg isotopic composition (gray band) is from (*10*). NK–Nankai, LA–Lesser Antilles, NI - Nicaragua, MA–Northern Marianas, AL–Central-Eastern Aleutians, SSI–South Sandwich Islands, NH–Northern Honshu, KA–Kamchatka, Mt. Baker–Cascades.

This trend extends toward more mantle-like Mg isotopic compositions in hotter subduction zones. Although warmer subduction zones are expected to promote greater Mg mobility and metasomatism (*54*), they do not produce the heaviest Mg isotopic signatures, such as in the North Cascades arc (*55*). This may reflect dilution of the heavy Mg isotopic signal in Type 1 melts through mixing with more abundant Type 2 melts generated in the hotter mantle wedge interior (*5*, *56*), or enhanced forearc dehydration that limits slab-derived inputs to the base of subarc mantle wedge, consistent with the lack of prominent FME enrichment in the Cascades (*31*, *57*).

The coldest arcs, including North Honshu and Kamchatka, deviate from the pattern and also have mantle-like δ^26^Mg values. At high slab thermal parameters, rapid convergence and cold slab might cool the base of the mantle wedge sufficiently to inhibit water-saturated melting at sub-arc depth (*3*). The subducting Hawaii-Emperor Seamount Chain could also affect Kamchatka arc magmas (*58*), although its role in Mg isotopes remains uncertain.

Subduction geometry could also influence arc δ^26^Mg (Fig. 6B). The highest δ^26^Mg arc magmas occur in the steepest subduction settings (i.e. SSI), suggesting a geometric focusing effect on deep hydrous melts. Since isotherms in the mantle wedge are largely parallel to the slab near the slab-mantle interface, the geometry of the water-saturated mantle melting zone can affect the location of the volcanic front. Deep melts rising dominantly sub-vertically may be dispersed over a longer horizontal distance, generating broader rear-arc volcanism in addition to the frontal arc (*59*).

Preservation of high δ^26^Mg signature from the slab-mantle interface requires chemically isolated melt extraction with minimal interaction with the overlying mantle wedge to prevent isotopic re-equilibration. This is supported by the short melt transport timescales calculated from U-series disequilibria and the preservation of slab-derived B and Os isotopic signatures in arc magma, despite their fast diffusion rates and compatibility in ultramafic rocks (*60*–*62*). Together, these observations favor a rapid, channelized melt ascent over reactive porous flow. Alternatively, ascent of hybridized mélange diapirs has been proposed to explain certain arc geochemical signatures, particularly B and Ba isotopes in the South Sandwich Islands (*16*, *62*–*64*). Distinguishing between these transport mechanisms remains a key challenge in constraining the origins of slab-derived signatures in arc magmas and the fate of subducted volatiles.

## MATERIALS AND METHODS

### Sample descriptions

#### Nicaragua

We analyzed 14 well-characterized samples from Nicaragua, including lava, scoria, and tephra hosted bombs from Cosigüina, San Cristobal, Telica, Cerro Negro, Momotombo, Granada, and Nejapa. These samples are basaltic to andesitic with SiO_2_ content of 47.2–58.9 wt.% and Mg# of 35.5–63.0. Major and trace element concentrations, and Sr-Nd-Pb isotope compositions of these samples are included in the comprehensive RU_CAGeochem dataset curated by Carr *et al.* (*65*) Oxygen isotopic composition of olivine and plagioclase phenocrysts hosted in these samples reveals a mantle source enriched with a water-rich, low δ^18^O component, likely a slab-derived fluid, in the Nicaragua subarc mantle (*66*). Lithium isotopic compositions of these samples were reported by (*67*, *68*).

#### South Sandwich Islands

We analyzed 27 samples from 12 major volcanic centers from South Sandwich Islands (SSI) arc. A compilation (including new analyses) of the whole rock major and trace element concentrations, Sr-Nd-Pb isotopic compositions, as well as a description of the general petrography of these samples were reported by Pearce *et al.* (*69*). Present day volcanism in the SSI arc was estimated to have initiated around 5 Ma (*70*). Our samples range from basaltic to andesitic, and can be classified into distinct low-K tholeiitic, tholeiitic, and calc-alkalic series (*69*). Boron concentration and isotopic composition of these samples indicate a strong contribution from hydrated forearc mantle (*62*). Qiao *et al.* (*16*) reported Mg isotopic compositions of similar SSI arc samples in the range of −0.25 to −0.06 ‰, agreeing well with our measurements. Boron isotopic compositions of these samples were reported by (*62*).

#### Central-eastern Aleutian

We analyzed 18 well-characterized lava samples from Central-Eastern section of the Aleutian arc, covering Shishaldin, Okmok, Recheschnoi, Yunaska, Seguam, and Kanaga volcanoes. These lavas are Pleistocene-recent, and their major and trace element concentrations, as well as Sr-Nd-Pb isotopes of the Central-Eastern Aleutian samples were reported by Jicha *et al.* (*71*) and Singer *et al.* (*72*) Our samples are all basaltic with SiO_2_ content ranging 47.9–50.9 wt %, and have Mg# from 47.9 to 73.8. Note that samples from Seguam occur in a region of fracture zone subduction, where influx of slab-derived fluids is likely abnormally high (*61*, *73*, *74*). Boron isotopic compositions of these samples were reported by (*61*).

#### Magnesium isotope analysis

Magnesium isotopic analyses were performed at the Non-Traditional Isotope Laboratory of the University of Washington, following previously established protocols (*75*), which are briefly summarized here. Whole rock powders were dissolved in a mixture of Optima-grade HF-HNO_3_-HCl in closed Savillex vessels on a hotplate, until no visible particulate residue was present in the solution. Sample solutions were subsequently evaporated to dryness and fluxed with 1 N HNO_3_ for column chemistry. We loaded solutions containing ∼10 μg of Mg onto the columns, and Mg was extracted and purified by two passes of elution with pre-cleaned Bio-Rad AG50W-X8 (200–400 mesh) cation exchange resin in 1 N HNO_3_. The purified Mg sample solutions were analyzed using a Nu Plasma II multicollector-ICPMS using the standard-sample-bracketing method (*75*).

#### Monte Carlo isotope mixing

To account for the range of MgO content and Mg isotopic composition of melts generated in the deep vs shallow mantle wedge, we use a forward Monte Carlo approach to estimate the proportion of melt originating from the slab-mantle interface region required to reproduce the Mg isotope composition of arc lavas. We generate 10,000 random mixtures of the two endmember melts. We use major element composition from experimentally produced melts at >3 GPa to represent Type 1 melt and 1–1.6 GPa to represent Type 2 melt (*3*, *8*, *47*). In each simulation, we randomly assign a mixing proportion between 0 to 1 and select one experiment from each group for MgO and SiO_2_ content. The δ^26^Mg for each endmember is randomly generated from a normal distribution with 0.00 ± 0.15 ‰ for melt 1 and − 0.25 ± 0.04 ‰ for melt 2. Then we compute the Mg isotopic composition of the mixture using binary mixing of the two generated endmembers. The results are presented in fig. S2.

We calculate an average δ^26^Mg for each subduction zone. Each arc average is calculated from an average of each volcanic center using samples with MgO content >5 wt % and standard deviation between volcanic centers. A synthetic mixture is permissible if its SiO_2_ content is <52 wt % and Mg isotopic composition falls within the δ^26^Mg range defined by the arc average ± 2SD, except for Mt. Baker which has an imposed range of ±0.05‰ to capture analytical uncertainty.

## Supplementary Materials

### This PDF file includes:

Figs. S1 and S2 Tables S1 to S3 Legend for data S1 References
