# Supplementary material for: Deep hydrous melting of the mantle wedge recorded by Mg isotopes
Source: Sci Adv. 2026 Oct 14;12(42):eaei6572. doi: 10.1126/sciadv.aei6572 (PMC13666000; doi:10.1126/sciadv.aei6572)
Supplement: Supplementary file 1 [file sciadv.aei6572_sm.pdf]

Science Advances
AAAS

# Supplementary Materials for

## Deep hydrous melting of the mantle wedge recorded by Mg isotopes

Winnie X. Fan *et al.*

Corresponding author: Winnie X. Fan, fanxuefe@uw.edu; Fang-Zhen Teng, fteng@uw.edu

*Sci. Adv.* **12**, eaei6572 (2026)
DOI: 10.1126/sciadv.aei6572

**The PDF file includes:**

Figs. S1 and S2
Tables S1 to S3
Legend for data S1
References

**Other Supplementary Material for this manuscript includes the following:**

Data S1

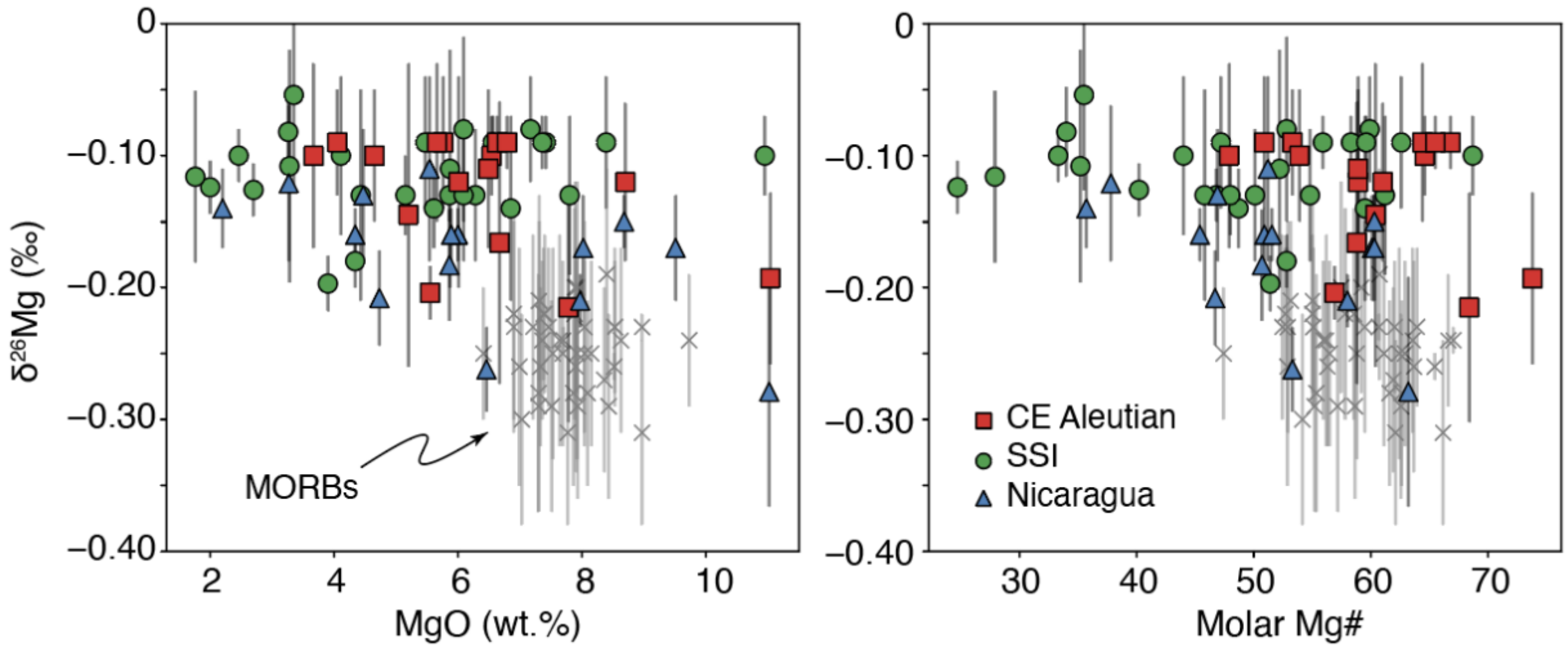


**Figure S1** Plots of $\delta^{26}Mg$ versus MgO content and molar Mg # for arc lava samples analyzed in this study. MORB data are from (*76*).

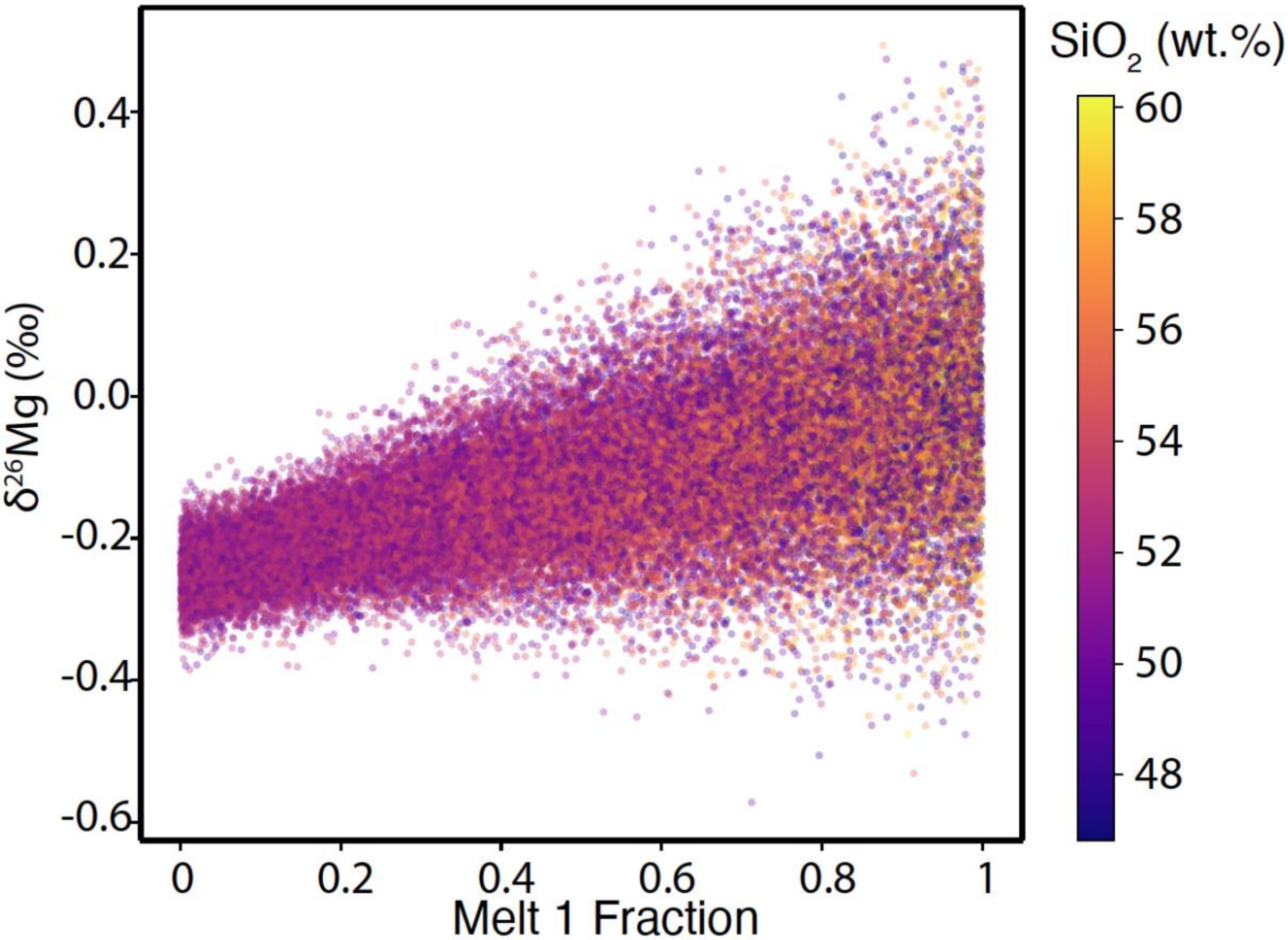


**Figure S2** Results of Monte Carlo mixing simulation plotted by simulated $\delta^{26}$Mg vs. fraction of deep water-saturated melt. Note that these mixing instances are not filtered, and all model results are included regardless of $SiO_2$ content.

**Table S1.**
Magnesium isotope composition of arc lavas analyzed in this study.

| Volcano | Sample | | $\delta^{26}$Mg (‰) | 2SD (‰) | $\delta^{25}$Mg (‰) | 2SD (‰) |
|---|---|---|---|---|---|---|
| **CE Aleutian** | | | | | | |
| Recheshnoi | CNU-50 | | −0.10 | 0.07 | −0.06 | 0.07 |
| | CNU-54b | | −0.16 | 0.07 | −0.07 | 0.12 |
| | | Replicate | −0.21 | 0.05 | −0.12 | 0.04 |
| | | **Average** | −0.19 | 0.04 | −0.12 | 0.04 |
| Yunaska | CS-22 | | −0.12 | 0.07 | −0.06 | 0.07 |
| | CS-24 | | −0.20 | 0.07 | −0.08 | 0.07 |
| | | Replicate | −0.22 | 0.05 | −0.10 | 0.04 |
| | | **Average** | −0.21 | 0.04 | −0.10 | 0.04 |
| Okmok | ID-20 | | −0.11 | 0.07 | −0.08 | 0.07 |
| | | Duplicate | −0.10 | 0.07 | −0.04 | 0.07 |
| | | **Average** | −0.11 | 0.05 | −0.06 | 0.05 |
| | ID-5 | | −0.12 | 0.07 | −0.08 | 0.07 |
| | OK-6 | | −0.12 | 0.07 | −0.02 | 0.07 |
| | | Duplicate | −0.07 | 0.07 | −0.03 | 0.07 |
| | | **Average** | −0.10 | 0.05 | −0.02 | 0.05 |
| Kanaga | KG-2 | | −0.12 | 0.07 | −0.03 | 0.06 |
| | | Duplicate | −0.17 | 0.05 | −0.07 | 0.04 |
| | | Replicate | −0.17 | 0.04 | −0.07 | 0.04 |
| | | **Average** | −0.16 | 0.03 | −0.06 | 0.03 |
| | KG-8 | | −0.13 | 0.07 | −0.04 | 0.07 |
| | | Duplicate | −0.06 | 0.07 | −0.06 | 0.07 |
| | | **Average** | −0.09 | 0.05 | −0.05 | 0.05 |
| Seguam | SB-87-32 | | −0.09 | 0.07 | −0.05 | 0.07 |
| | SB-87-34 | | −0.09 | 0.07 | −0.01 | 0.07 |
| | SB-87-35 | | −0.09 | 0.07 | −0.05 | 0.07 |
| | | Duplicate | −0.12 | 0.07 | −0.06 | 0.07 |
| | | **Average** | −0.10 | 0.05 | −0.06 | 0.05 |
| | SB-87-36 | | −0.18 | 0.07 | −0.07 | 0.07 |
| | | Replicate | −0.22 | 0.05 | −0.07 | 0.04 |
| | | **Average** | −0.19 | 0.04 | −0.08 | 0.03 |

**Table S1** (Continued)

| Volcano | Sample | | δ$^{26}$Mg (‰) | 2SD (‰) | δ$^{25}$Mg (‰) | 2SD (‰) |
|---|---|---|---|---|---|---|
| | SB-87-57 | | −0.15 | 0.05 | −0.07 | 0.04 |
| | | Duplicate | −0.15 | 0.04 | −0.08 | 0.04 |
| | | **Average** | −0.15 | 0.03 | −0.07 | 0.03 |
| | SB-87-8 | | −0.10 | 0.07 | −0.03 | 0.07 |
| | SB-88-6 | | −0.09 | 0.07 | −0.07 | 0.07 |
| | SB-88-19 | | −0.11 | 0.07 | −0.05 | 0.07 |
| Shishaldin | Shishaldin-15 | | −0.10 | 0.07 | −0.03 | 0.07 |
| | | Duplicate | −0.14 | 0.07 | −0.10 | 0.07 |
| | | **Average** | −0.12 | 0.05 | −0.07 | 0.05 |
| **Nicaragua** | | | | | | |
| Cerro Negro | CN1 | | −0.20 | 0.09 | −0.10 | 0.08 |
| | | Duplicate | −0.23 | 0.07 | −0.04 | 0.12 |
| | | Replicate | −0.21 | 0.05 | −0.11 | 0.04 |
| | | **Average** | −0.21 | 0.04 | −0.10 | 0.04 |
| | CN10 | | −0.16 | 0.07 | −0.06 | 0.07 |
| Granada | GR5 | | −0.21 | 0.09 | −0.14 | 0.08 |
| Nejapa | NE3 | | −0.11 | 0.07 | −0.06 | 0.07 |
| | NE5 | | −0.17 | 0.07 | −0.09 | 0.07 |
| | NE6 | | −0.15 | 0.07 | −0.10 | 0.07 |
| | NE202 | | −0.17 | 0.07 | −0.07 | 0.07 |
| | NE203 | | −0.31 | 0.09 | −0.13 | 0.08 |
| | | Replicate | −0.25 | 0.05 | −0.12 | 0.04 |
| | | **Average** | −0.26 | 0.04 | −0.12 | 0.04 |
| Momotombo | Mt-101 | | −0.12 | 0.07 | −0.02 | 0.07 |
| | | Duplicate | −0.14 | 0.07 | −0.10 | 0.07 |
| | | **Average** | −0.13 | 0.05 | −0.06 | 0.05 |
| Cosiguina | Cos-9a | | −0.15 | 0.07 | −0.06 | 0.07 |
| | | Duplicate | −0.13 | 0.07 | −0.11 | 0.07 |
| | | **Average** | −0.14 | 0.05 | −0.08 | 0.05 |
| San | SC2 | | −0.15 | 0.09 | −0.05 | 0.08 |
| | | Duplicate | −0.18 | 0.07 | −0.11 | 0.12 |
| | | **Average** | −0.17 | 0.06 | −0.07 | 0.07 |

**Table S1** (Continued)

| Volcano | Sample | | $\delta^{26}Mg$ (‰) | 2SD (‰) | $\delta^{25}Mg$ (‰) | 2SD (‰) |
|---|---|---|---|---|---|---|
| | SC3 | | −0.04 | 0.07 | −0.03 | 0.07 |
| | | Duplicate | −0.07 | 0.07 | −0.06 | 0.12 |
| | | Replicate | −0.12 | 0.05 | −0.04 | 0.04 |
| | | **Average** | −0.09 | 0.04 | −0.04 | 0.04 |
| Telica | TE3 | | −0.16 | 0.09 | −0.08 | 0.08 |
| | TE6 | | −0.25 | 0.07 | −0.06 | 0.07 |
| | | Duplicate | −0.28 | 0.07 | −0.16 | 0.12 |
| | | Replicate | −0.26 | 0.05 | −0.14 | 0.04 |
| | | **Average** | −0.26 | 0.04 | −0.12 | 0.04 |
| **South Sandwich Islands** | | | | | | |
| Vindication | SS 12.7 | | −0.09 | 0.07 | −0.02 | 0.07 |
| | SSV 2.1 | | −0.14 | 0.07 | −0.09 | 0.08 |
| Bristol | SS 15.2 | | −0.06 | 0.06 | 0.00 | 0.07 |
| | | Duplicate | −0.11 | 0.07 | −0.08 | 0.07 |
| | | **Average** | −0.08 | 0.05 | −0.04 | 0.05 |
| Bellingshausen | SS 17.10 | | −0.07 | 0.06 | −0.04 | 0.07 |
| | | Duplicate | −0.13 | 0.04 | −0.04 | 0.04 |
| | | **Average** | −0.11 | 0.03 | −0.04 | 0.04 |
| | SSB 11.1 | | −0.09 | 0.07 | −0.05 | 0.08 |
| | | Duplicate | −0.11 | 0.07 | −0.03 | 0.07 |
| | | **Average** | −0.10 | 0.05 | −0.04 | 0.05 |
| Saunders | SS 18.11 | | −0.09 | 0.06 | −0.07 | 0.07 |
| | SS 18.18 | | −0.13 | 0.06 | −0.06 | 0.07 |
| | | Duplicate | −0.13 | 0.06 | −0.05 | 0.07 |
| | | Duplicate | −0.15 | 0.07 | −0.09 | 0.06 |
| | | **Average** | −0.13 | 0.04 | −0.07 | 0.04 |
| | SS 18.3 | | −0.13 | 0.06 | −0.06 | 0.07 |
| | SS 18.7 | | −0.09 | 0.06 | −0.05 | 0.07 |
| | SS 21.3 | | −0.08 | 0.06 | −0.05 | 0.07 |

**Table S1** (Continued)

| Volcano | Sample | | δ$^{26}$Mg (‰) | 2SD (‰) | δ$^{25}$Mg (‰) | 2SD (‰) |
|---|---|---|---|---|---|---|
| | SS 21.4 | | −0.12 | 0.06 | −0.08 | 0.07 |
| | | Duplicate | −0.14 | 0.07 | −0.04 | 0.07 |
| | | **Average** | −0.13 | 0.05 | −0.06 | 0.05 |
| | SSS 5.4 | | −0.14 | 0.07 | −0.09 | 0.08 |
| | SSS 15.1 | | −0.13 | 0.07 | −0.07 | 0.08 |
| Cook | SSK 1.1 | | −0.15 | 0.07 | −0.05 | 0.08 |
| | | Duplicate | −0.12 | 0.07 | −0.02 | 0.12 |
| | | Replicate | −0.12 | 0.07 | −0.06 | 0.08 |
| | | **Average** | −0.13 | 0.04 | −0.05 | 0.05 |
| Candlemas | SSC 41.1 | | −0.20 | 0.07 | −0.12 | 0.06 |
| | | Duplicate | −0.18 | 0.04 | −0.10 | 0.04 |
| | | **Average** | −0.19 | 0.04 | −0.11 | 0.04 |
| | SSC 35.1 | | −0.13 | 0.07 | −0.02 | 0.08 |
| | | Duplicate | −0.10 | 0.07 | −0.12 | 0.12 |
| | | **Average** | −0.11 | 0.05 | −0.05 | 0.07 |
| | SS 82.2 | | −0.10 | 0.07 | −0.08 | 0.08 |
| | SS 78.1 | | −0.09 | 0.07 | 0.10 | 0.12 |
| | | Duplicate | −0.09 | 0.07 | −0.02 | 0.06 |
| | | Duplicate | −0.12 | 0.04 | −0.06 | 0.04 |
| | | **Average** | −0.11 | 0.03 | −0.04 | 0.03 |
| Freezeland | SSF 2.2 | | −0.20 | 0.07 | −0.06 | 0.06 |
| Leskov | SSL 4.2 | | −0.21 | 0.07 | −0.10 | 0.08 |
| | | Duplicate | −0.19 | 0.07 | −0.07 | 0.12 |
| | | **Average** | −0.20 | 0.05 | −0.09 | 0.07 |
| Visokoi | SSW 2.1 | | −0.13 | 0.07 | −0.06 | 0.08 |
| | SSW 3.1 | | −0.10 | 0.07 | −0.07 | 0.08 |
| Montague | SS 21.5 | | −0.09 | 0.07 | −0.06 | 0.08 |
| | | Duplicate | −0.10 | 0.07 | −0.03 | 0.07 |
| | | **Average** | −0.10 | 0.05 | −0.04 | 0.05 |

**Table S1** (Continued)

| Volcano | Sample | | $\delta^{26}Mg$ (‰) | 2SD (‰) | $\delta^{25}Mg$ (‰) | 2SD (‰) |
|---|---|---|---|---|---|---|
| Thule | SST 7.1 | | −0.13 | 0.07 | −0.04 | 0.08 |
| | | Duplicate | −0.14 | 0.07 | −0.12 | 0.12 |
| | | **Average** | −0.13 | 0.05 | −0.06 | 0.07 |
| | SST 4.1 | | −0.10 | 0.07 | −0.06 | 0.08 |
| | | Duplicate | −0.08 | 0.09 | −0.04 | 0.08 |
| | | Duplicate | −0.12 | 0.07 | −0.02 | 0.12 |
| | | Duplicate | −0.09 | 0.04 | −0.04 | 0.04 |
| | | **Average** | −0.10 | 0.03 | −0.04 | 0.03 |
| Zavadoski | SS 19.2 | | −0.08 | 0.06 | −0.06 | 0.07 |
| | SS 19.5 | | −0.13 | 0.06 | −0.05 | 0.07 |

Duplicate indicates repeat analysis on the same Mg column cut; replicate indicates repeat sample dissolution, column chemistry and analysis. Average of repeat analyses is the inverse-variance weighted mean.

**Table S2** Magnesium isotopic compositions of reference materials analyzed during this study.

| Standard Name | $\delta^{26}Mg$ (‰) | 2SD (‰) | $\delta^{25}Mg$ (‰) | 2SD (‰) |
|---|---|---|---|---|
| **BHVO-1** | −0.24 | 0.04 | −0.13 | 0.04 |
| | −0.21 | 0.05 | −0.09 | 0.04 |
| **Average (n=2)** | **−0.23** | **0.03** | **−0.11** | **0.03** |
| **Recommended value[a]** | **−0.22** | **0.07** | **−0.13** | **0.03** |
| | | | | |
| **Seawater** | −0.83 | 0.07 | −0.38 | 0.07 |
| | −0.75 | 0.07 | −0.36 | 0.08 |
| | −0.84 | 0.07 | −0.42 | 0.07 |
| | −0.79 | 0.07 | −0.44 | 0.07 |
| | −0.77 | 0.07 | −0.37 | 0.12 |
| | −0.84 | 0.05 | −0.44 | 0.04 |
| **Average (n = 6)** | **−0.81** | **0.03** | **−0.42** | **0.03** |
| **Recommended value[a]** | **−0.83** | **0.09** | **−0.43** | **0.06** |
| | | | | |
| **JG-1** | −0.23 | 0.04 | −0.10 | 0.05 |
| **Recommended value[a]** | **−0.31** | **0.07** | **−0.16** | **0.03** |
| | | | | |
| **Phlogopite** | −1.32 | 0.04 | −0.72 | 0.04 |
| **Recommended value[a]** | **−1.37** | **0.07** | **−0.72** | **0.05** |
| | | | | |
| **San Carlos Olivine** | −0.27 | 0.07 | −0.13 | 0.07 |
| | −0.24 | 0.07 | −0.14 | 0.07 |
| | −0.27 | 0.06 | −0.14 | 0.07 |
| | −0.25 | 0.07 | −0.07 | 0.08 |
| | −0.24 | 0.07 | −0.16 | 0.08 |
| | −0.29 | 0.07 | −0.15 | 0.07 |
| | −0.26 | 0.07 | −0.11 | 0.07 |
| | −0.26 | 0.07 | −0.15 | 0.07 |
| | −0.30 | 0.07 | −0.09 | 0.12 |
| | −0.27 | 0.07 | −0.15 | 0.12 |
| | −0.26 | 0.09 | −0.10 | 0.08 |
| **Average (n = 11)** | **−0.26** | **0.02** | **−0.13** | **0.02** |
| **Recommended value[b]** | **−0.24** | **0.03** | **−0.12** | **0.02** |

a – Teng et al. (2017)(82); b – Hu et al. (2016)(83).

**Table S3** Calculated average $\delta^{26}$Mg and selected subduction parameters plotted in Figure 6.

| Arc/Arc segment | N volcano | Average $\delta^{26}$Mg (‰) | 2SD (‰) | Thermal parameter/100 (km) | Arc-trench distance (km) |
|---|---|---|---|---|---|
| C-E Aleut | 6 | −0.15 | 0.07 | 25.4 | 204 |
| Kamchatka | 6 | −0.29 | 0.08 | 54.1 | 227 |
| Mt Baker | 1 | −0.28 | | 1.3 | 345 |
| Lesser Antilles | 8 | −0.22 | 0.06 | 10.75 | 224 |
| N Mariana | 3 | −0.16 | 0.09 | 19.9 | 198 |
| SSI | 12 | −0.11 | 0.03 | 28.6 | 161 |
| Nicaragua | 7 | −0.20 | 0.07 | 11 | 203 |
| Nankai | 8 | −0.21 | 0.07 | 4.5 | 340 |
| N Honshu | 3 | −0.19 | 0.09 | 50.7 | 319 |

Average includes sample with MgO content >5 wt. % and is first averaged at each volcanic center. Thermal parameter and arc-trench distance are from (*53*), except for Nankai arc-trench distance is from (*77*).

**Data S1. (separate file)**

Major elements, trace elements and radiogenic isotopes data of arc magma samples compiled with Mg isotopic compositions.

## REFERENCES

1. T. L. Grove, C. B. Till, M. J. Krawczynski, The role of $H_2O$ in subduction zone magmatism. *Annu. Rev. Earth Planet. Sci.* **40**, 413–439 (2012).

2. W. P. Leeman, Old/new subduction zone paradigms as seen from the Cascades. *Front. Earth Sci.* **8**, 10.3389/feart.2020.535879 (2020).

3. C. B. Till, T. L. Grove, A. C. Withers, The beginnings of hydrous mantle wedge melting. *Contrib. Mineral. Petrol.* **163**, 669–688 (2012).

4. J. Wang, E. Takahashi, X. Xiong, L. Chen, L. Li, T. Suzuki, M. J. Walter, The water-saturated solidus and second critical endpoint of peridotite: implications for magma genesis within the mantle *Wedge*. *J. Geophys. Res. Space Phys.* **125**, e2020JB019452 (2020).

5. P. C. England, R. F. Katz, Melting above the anhydrous solidus controls the location of volcanic arcs. *Nature* **467**, 700–703 (2010).

6. P. C. England, R. F. Katz, Global systematics of arc volcano position. *Nature* **468**, E6–E7 (2010).

7. T. L. Grove, C. B. Till, E. Lev, N. Chatterjee, E. Médard, Kinematic variables and water transport control the formation and location of arc volcanoes. *Nature* **459**, 694–697 (2009).

8. T. L. Grove, C. B. Till, $H_2O$-rich mantle melting near the slab–wedge interface. *Contrib. Mineral. Petrol.* **174**, 80 (2019).

9. C. B. Till, A review and update of mantle thermobarometry for primitive arc magmas. *Am. Mineral.* **102**, 913–947 (2017).

10. F.-Z. Teng, M. Wadhwa, R. T. Helz, Investigation of magnesium isotope fractionation during basalt differentiation: Implications for a chondritic composition of the terrestrial mantle. *Earth Planet. Sci. Lett.* **261**, 84–92 (2007).

11. Y. Hu, F.-Z. Teng, D. A. Ionov, Magnesium isotopic composition of metasomatized upper sub-arc mantle and its implications to Mg cycling in subduction zones. *Geochim. Cosmochim. Acta* **278**, 219–234 (2020).

12. G. Easthouse, W. Hoover, F.-Z. Teng, C. Condit, C. Pike, Z.-Z. Wang, A. Berg, P. Wynn, L. Woods, E. Poulaki, Formation of talc in the subduction interface: Mg isotopes demonstrate Mg loss over Si gain. *Geology* **53**, 398–403 (2025).

13. W.-Y. Li, F.-Z. Teng, Y. Xiao, Magnesium isotope record of fluid metasomatism along the slab-mantle interface in subduction zones. *Geochim. Cosmochim. Acta* **237**, 312–319 (2018).

14. P. A. E. Pogge von Strandmann, R. Dohmen, H. R. Marschall, J. C. Schumacher, T. Elliott, Extreme magnesium isotope fractionation at outcrop scale records the mechanism and rate at which reaction fronts advance. *J. Petrol.* **56**, 33–58 (2015).

15. C. R. Soderman, S. Matthews, O. Shorttle, M. G. Jackson, J. M. D. Day, V. Kamenetsky, H. M. Williams, Global oceanic basalt sources and processes viewed through combined Fe and Mg stable isotopes. *Earth Planet. Sci. Lett.* **638**, 118749 (2024).

16. X.-Y. Qiao, J.-W. Xiong, Y.-X. Chen, J. C. M. De Hoog, J. Pearce, F. Huang, Z.-F. Zhao, K. Chen, Magnesium and boron isotope evidence for the generation of arc magma through serpentinite mélange melting. *Natl. Sci. Rev.* **12**, nwae363 (2024).

17. F.-Z. Teng, Y. Hu, C. Chauvel, Magnesium isotope geochemistry in arc volcanism. *Proc. Natl. Acad. Sci. U.S.A.* **113**, 7082–7087 (2016).

18. X.-N. Liu, R. C. Hin, C. D. Coath, M. Van Soest, E. Melekhova, T. Elliott, Equilibrium olivine-melt Mg isotopic fractionation explains high δ26Mg values in arc lavas. *Geochem. Persp. Let.* **22**, 42–47 (2022).

19. S.-G. Li, W. Yang, S. Ke, X. Meng, H. Tian, L. Xu, Y. He, J. Huang, X.-C. Wang, Q. Xia, W. Sun, X. Yang, Z.-Y. Ren, H. Wei, Y. Liu, F. Meng, J. Yan, Deep carbon cycles constrained by a large-scale mantle Mg isotope anomaly in eastern China. *Natl. Sci. Rev.* **4**, 111–120 (2017).

20. F.-Z. Teng, N. Dauphas, R. T. Helz, S. Gao, S. Huang, Diffusion-driven magnesium and iron isotope fractionation in Hawaiian olivine. *Earth Planet. Sci. Lett.* **308**, 317–324 (2011).

21. F. M. Richter, E. B. Watson, R. A. Mendybaev, F.-Z. Teng, P. E. Janney, Magnesium isotope fractionation in silicate melts by chemical and thermal diffusion. *Geochim. Cosmochim. Acta* **72**, 206–220 (2008).

22. B. da Silva Ricardo, M. Oeser, K. J. Lynn, N. R. Bennett, X. Chu, G. Henderson, Y. Liu, S. Weyer, C. K. Sio, Chemical and isotopic fractionation during melt inclusion formation. *Geology* **53**, 375–379 (2025).

23. X.-J. Wang, L.-H. Chen, T. Hanyu, Y. Zhong, J.-H. Shi, X.-W. Liu, H. Kawabata, G. Zeng, L.-W. Xie, Magnesium isotopic fractionation during basalt differentiation as recorded by evolved magmas. *Earth Planet. Sci. Lett.* **565**, 116954 (2021).

24. Y. Sun, F.-Z. Teng, T. O. Rooney, K.-N. Pang, Z.-Z. Wang, Magnesium isotope behavior during titanomagnetite fractionation in basaltic lavas. *Chem. Geol.* **655**, 122088 (2024).

25. J. A. Walker, Volcanic rocks from the Nejapa and Granada cinder cone alignments, Nicaragua, Central America. *J. Petrol.* **25**, 299–342 (1984).

26. J. A. Walker, M. J. Carr, M. D. Feigenson, R. I. Kalamarides, The petrogenetic significance of interstratified high- and low-Ti basalts in central Nicaragua. *J. Petrol.* **31**, 1141–1164 (1990).

27. D. R. Avellán, J. L. Macías, N. Pardo, T. Scolamacchia, D. Rodriguez, Stratigraphy, geomorphology, geochemistry and hazard implications of the Nejapa Volcanic Field, western Managua, Nicaragua. *J. Volcanol. Geotherm. Res.* **213-214**, 51–71 (2012).

28. E. Gazel, K. E. Flores, M. J. Carr, Architectural and tectonic control on the segmentation of the Central American volcanic arc. *Annu. Rev. Earth Planet. Sci.* **49**, 495–521 (2021).

29. E. Melekhova, J. Blundy, Generation of mantle-derived basaltic andesites in volcanic arcs. *Earth Planet. Sci. Lett.* **641**, 118791 (2024).

30. E. K. Mullen, D. Weis, Evidence for trench-parallel mantle flow in the northern Cascade Arc from basalt geochemistry. *Earth Planet. Sci. Lett.* **414**, 100–107 (2015).

31. J. G. Ryan, C. Chauvel, “The Subduction-Zone Filter and the Impact of Recycled Materials on the Evolution of the Mantle” in *Treatise on Geochemistry* (Elsevier, 2014), pp. 479–508.

32. F. Deschamps, S. Guillot, M. Godard, M. Andreani, K. Hattori, Serpentinites act as sponges for fluid-mobile elements in abyssal and subduction zone environments. *Terra Nova* **23**, 171–178 (2011).

33. F. Deschamps, M. Godard, S. Guillot, K. Hattori, Geochemistry of subduction zone serpentinites: A review. *Lithos* **178**, 96–127 (2013).

34. L. Du, Y. Zhang, H. Zhu, Z. Zhang, Y. An, C. Yuan, Z. Huang, X. Li, X. Long, Different effects of serpentinite-derived and slab-derived fluids on arc magmatism in the Chinese Eastern Tianshan: evidence from Mg–B isotopic systematics. *J. Geol. Soc. London* **180**, 10.1144/jgs2023-011 (2023).

35. P.-P. Liu, F.-Z. Teng, H. J. B. Dick, M.-F. Zhou, S.-L. Chung, Magnesium isotopic composition of the oceanic mantle and oceanic Mg cycling. *Geochim. Cosmochim. Acta* **206**, 151–165 (2017).

36. X. Deng, Y.-X. Chen, W. Wang, Y. Li, Z. Xiao, Z. Wu, Heavy magnesium isotopic signatures in arc lavas may be attributed to dehydration of subducting hydrated mantle. *Commun. Earth Environ.* **5**, 299 (2024).

37. Y. Wang, J. Deng, R. Liao, L. Chen, D. Li, H. Liu, W. Sun, Magnesium isotopic composition of the Mariana forearc serpentinite: Implications for Mg isotopic composition of the mantle wedge and Mg isotopic fractionation during mantle wedge serpentinization. *Chem. Geol.* **624**, 121428 (2023).

38. S. G. Nielsen, F. Klein, H. R. Marschall, P. A. E. Pogge Von Strandmann, M. Auro, Magnesium isotope fractionation processes during seafloor serpentinization and implications for serpentinite subduction. *Solid Earth* **15**, 1143–1154 (2024).

39. Y.-R. Qu, S.-A. Liu, H. Gamaleldien, Insights into subduction-zone fluid-rock interactions and carbon cycling from magnesium isotopes of subducted ophiolitic mélanges in the Arabian-Nubian shield. *Geochem. Geophys. Geosyst.* **26**, e2024GC011918 (2025).

40. S. M. Peacock, K. Wang, On the stability of talc in subduction zones: a possible control on the maximum depth of decoupling between the subducting plate and mantle wedge. *Geophys. Res. Lett.* **48**, e2021GL094889 (2021).

41. M. Scambelluri, E. Cannaò, M. Gilio, The water and fluid-mobile element cycles during serpentinite subduction. A review. *Eur. J. Mineral.* **31**, 405–428 (2019).

42. S. Lakey, J. Hermann, An experimental study of chlorite stability in varied subduction zone lithologies with implications for fluid production, melting, and diapirism in chlorite-rich mélange rocks. *J. Petrol.* **63**, egac029 (2022).

43. T. L. Grove, N. Chatterjee, S. W. Parman, E. Médard, The influence of H2O on mantle wedge melting. *Earth Planet. Sci. Lett.* **249**, 74–89 (2006).

44. Y. Tatsumi, Formation of the volcanic front in subduction zones. *Geophys. Res. Lett.* **13**, 717–720 (1986).

45. K. H. Hattori, S. Guillot, Volcanic fronts form as a consequence of serpentinite dehydration in the forearc mantle wedge. *Geology* **31**, 525–528 (2003).

46. P. Kelemen, K. Hanghøj, A. R. Greene, "One view of the geochemistry of subduction-related magmatic arcs, with an emphasis on primitive andesite and lower crust" in *Treatise on Geochemistry* (Elsevier, 2014; https://linkinghub.elsevier.com/retrieve/pii/B9780080959757005167), pp. 471–515.

47. A. L. Mitchell, T. L. Grove, Melting the hydrous, subarc mantle: the origin of primitive andesites. *Contrib. Mineral. Petrol.* **170**, 13 (2015).

48. G. A. Gaetani, T. L. Grove, The influence of water on melting of mantle peridotite. *Contrib. Mineral. Petrol.* **131**, 323–346 (1998).

49. H. Kawakatsu, S. Watada, Seismic evidence for deep-water transportation in the mantle. *Science* **316**, 1468–1471 (2007).

50. R. An, Y. He, S. Ke, G. Zhao, A. Sun, Y. Wang, H. Xu, Heavy magnesium isotope evidence for a key role of serpentinite-derived fluids in arc magmatism. *J. Earth Sci.* **36**, 2798–2802 (2025).

51. S. J. Turner, C. H. Langmuir, An alternative to the igneous crust fluid + sediment melt paradigm for arc lava geochemistry. *Sci. Adv.* **10**, eadg6482 (2024).

52. S. J. Turner, C. H. Langmuir, Sediment and ocean crust both melt at subduction zones. *Earth Planet. Sci. Lett.* **584**, 117424 (2022).

53. E. M. Syracuse, P. E. van Keken, G. A. Abers, The global range of subduction zone thermal models. *Phys. Earth Planet. In.* **183**, 73–90 (2010).

54. A. Okamoto, R. Oyanagi, Si- versus Mg-metasomatism at the crust–mantle interface: insights from experiments, natural observations and geochemical modeling. *Prog. Earth Planet. Sci.* **10**, 39 (2023).

55. A. W. Brewer, F.-Z. Teng, E. Mullen, Magnesium isotopes as a tracer of crustal materials in volcanic arc magmas in the northern Cascade arc. *Front. Earth Sci.* **6**, 21 (2018).

56. P. E. van Keken, B. Kiefer, S. M. Peacock, High-resolution models of subduction zones: Implications for mineral dehydration reactions and the transport of water into the deep mantle. *Geochem. Geophys. Geosyst.* **3**, 1056 (2002).

57. W. P. Leeman, S. Tonarini, L. H. Chan, L. E. Borg, Boron and lithium isotopic variations in a hot subduction zone—the southern Washington Cascades. *Chem. Geol.* **212**, 101–124 (2004).

58. Y. Shu, S. G. Nielsen, V. Le Roux, G. Wörner, J. Blusztajn, M. Auro, Sources of dehydration fluids underneath the Kamchatka arc. *Nat. Commun.* **13**, 4467 (2022).

59. K. M. Haase, M. Regelous, C. Beier, A. A. P. Koppers, Slab steepening and rapid mantle wedge replacement during back-arc rifting in the New Hebrides. *Nat. Commun.* **15**, 6070 (2024).

60. T. Elliott, M. Spiegelman, “Melt Migration in Oceanic Crustal Production: A U-Series Perspective” in *Treatise on Geochemistry* (Elsevier, 2014), pp. 543–581.

61. O. Jensen, R. Waldman, G. Yogodzinski, M. Bizimis, J. Ryan, W. Leeman, H. Scher, K. Hoernle, Boron isotopes identify deep-slab serpentinite in the source of Aleutian arc magma. *Geology* **53**, 114–118 (2025).

62. S. Tonarini, W. P. Leeman, P. T. Leat, Subduction erosion of forearc mantle wedge implicated in the genesis of the South Sandwich Island (SSI) arc: Evidence from boron isotope systematics. *Earth Planet. Sci. Lett.* **301**, 275–284 (2011).

63. Y. Shu, S. G. Nielsen, V. Le Roux, D. S. Ramos, J. Blusztajn, M. Auro, P. T. Leat, T. J. Horner, Mélange dehydration and melting beneath South Sandwich Islands arc. *Nat. Commun.* **16**, 1440 (2025).

64. S. G. Nielsen, H. R. Marschall, Geochemical evidence for mélange melting in global arcs. *Sci. Adv.* **3**, e1602402 (2017).

65. M. J. Carr, M. D. Feigenson, L. L. Bolge, J. A. Walker, E. Gazel, RU_CAGeochem, a database and sample repository for Central American volcanic rocks at Rutgers University. *Geosci. Data J.* **1**, 43–48 (2014).

66. J. M. Eiler, M. J. Carr, M. Reagan, E. Stolper, Oxygen isotope constraints on the sources of Central American arc lavas. *Geochem. Geophys. Geosyst.* **6**, Q07007 (2005).

67. L. H. Chan, W. P. Leeman, C.-F. You, Lithium isotopic composition of Central American Volcanic Arc lavas: implications for modification of subarc mantle by slab-derived fluids. *Chem. Geol.* **160**, 255–280 (1999).

68. L. H. Chan, W. P. Leeman, C.-F. You, Lithium isotopic composition of Central American volcanic arc lavas: implications for modification of subarc mantle by slab-derived fluids: correction. *Chem. Geol.* **182**, 293–300 (2002).

69. J. A. Pearce, P. E. Baker, P. K. Harvey, I. W. Luff, Geochemical evidence for subduction fluxes, mantle melting and fractional crystallization beneath the South Sandwich Island arc. *J. Petrol.* **36**, 1073–1109 (1995).

70. J. A. Pearce, A. R. Hastie, P. T. Leat, I. W. Dalziel, L. A. Lawver, P. F. Barker, I. L. Millar, T. L. Barry, R. E. Bevins, Composition and evolution of the Ancestral South Sandwich Arc: Implications for the flow of deep ocean water and mantle through the Drake Passage Gateway. *Global Planet. Change* **123**, 298–322 (2014).

71. B. R. Jicha, B. S. Singer, J. G. Brophy, J. H. Fournelle, C. M. Johnson, B. L. Beard, T. J. Lapen, N. J. Mahlen, Variable impact of the subducted slab on Aleutian island arc magma sources: evidence from Sr, Nd, Pb, and Hf isotopes and trace element abundances. *J. Petrol.* **45**, 1845–1875 (2004).

72. B. S. Singer, B. R. Jicha, W. P. Leeman, N. W. Rogers, M. F. Thirlwall, J. Ryan, K. E. Nicolaysen, Along-strike trace element and isotopic variation in Aleutian Island arc basalt: Subduction melts sediments and dehydrates serpentine. *J. Geophys. Res. Solid Earth* **112**, B06206 (2007).

73. V. C. Manea, W. P. Leeman, T. Gerya, M. Manea, G. Zhu, Subduction of fracture zones controls mantle melting and geochemical signature above slabs. *Nat. Commun.* **5**, 5095 (2014).

74. E. Rojas-Kolomiets, O. Jensen, M. Bizimis, G. Yogodzinski, L. Ackerman, Serpentinite fluids and slab-melting in the Aleutian arc: Evidence from molybdenum isotopes and boron systematics. *Earth Planet. Sci. Lett.* **603**, 117970 (2023).

75. F.-Z. Teng, W. Yang, Comparison of factors affecting the accuracy of high-precision magnesium isotope analysis by multi-collector inductively coupled plasma mass spectrometry. *Rapid Commun. Mass Spectrom.* **28**, 19–24 (2014).

76. F.-Z. Teng, W.-Y. Li, S. Ke, B. Marty, N. Dauphas, S. Huang, F.-Y. Wu, A. Pourmand, Magnesium isotopic composition of the Earth and chondrites. *Geochim. Cosmochim. Acta* **74**, 4150–4166 (2010).

77. W. Zhang, H. Kitagawa, F. Huang, Magnesium isotope composition of volcanic rocks from cold and warm subduction zones: Implications for the recycling of subducted serpentinites and carbonates. *Geochim. Cosmochim. Acta* **391**, 158–176 (2025).

78. Esri, Maxar, Earthstar Geographics, and the GIS User Community. World Imagery [Basemap]. *ArcGIS Online* (2026).

79. R. K. Workman, S. R. Hart, Major and trace element composition of the depleted MORB mantle (DMM). *Earth Planet. Sci. Lett.* **231**, 53–72 (2005).

80. W. P. Leeman, "Boron and other fluid-mobile elements in volcanic arc lavas: implications for subduction processes" in *Geophysical Monograph Series*, G. E. Bebout, D. W. Scholl, S. H. Kirby, J. P. Platt, Eds. (American Geophysical Union, Washington, D.C., 1996; http://doi.wiley.com/10.1029/GM096p0269), pp. 269–276.

81. M. Scambelluri, T. Pettke, E. Cannaò, Fluid-related inclusions in Alpine high-pressure peridotite reveal trace element recycling during subduction-zone dehydration of serpentinized mantle (Cima di Gagnone, Swiss Alps). *Earth Planet. Sci. Lett.* **429**, 45–59 (2015).

82. F.-Z. Teng, Magnesium Isotope Geochemistry. *Rev. Mineral. Geochem.* **82**, 219–287 (2017).

83. Y. Hu, M. D. Harrington, Y. Sun, Z. Yang, J. Konter, F.-Z. Teng, Magnesium isotopic homogeneity of San Carlos olivine: a potential standard for Mg isotopic analysis by multi-collector inductively coupled plasma mass spectrometry. *Rapid Commun. Mass Spectrom.* **30**, 2123–2132 (2016).
